# Work-related risk factors for sciatica leading to hospitalization

**DOI:** 10.1038/s41598-019-42597-w

**Published:** 2019-04-25

**Authors:** Ulla Euro, Markku Heliövaara, Rahman Shiri, Paul Knekt, Harri Rissanen, Arpo Aromaa, Jaro Karppinen

**Affiliations:** 1 Medical Research Center Oulu, Oulu University Hospital and University of Oulu, Oulu, Finland; 2 Center for Life Course Health Research, University of Oulu, Oulu, Finland; 3 National Institute for Health and Welfare, Department of Health, Helsinki, Finland; 4 Finnish Institute of Occupational Health, Helsinki, Finland; 5 National Institute of Health and Welfare, Department of Public Health Solutions, Helsinki, Finland; 6 Finnish Institute of Occupational Health, Oulu, Finland

**Keywords:** Medical research, Risk factors

## Abstract

The aim of this study was to assess the effects of the general strenuousness of work and various physical exposures on the risk of hospitalization for sciatica. The study population consisted of Finns aged 30 to 59 who had participated in a national health examination survey in 1978–80 (N = 3891). The participants were followed up until the end of 2011 and information on work-related determinants was acquired by a questionnaire. After adjustment for confounders, sedentary work involving handling fairly heavy objects/physically light work (HR 1.57; 95% CI 1.05–2.34), lifting or carrying heavy objects (2.10; 1.35–3.26) and exposure to whole-body vibration (1.61; 0.95–2.72) predicted sciatica, whereas heavier workloads appeared to reduce its risk (0.48; 0.26–0.89). There was an interaction between body mass index and exposure to whole-body vibration for the risk of sciatica. Overweight (1.94; 0.96–3.93) and obese (3.50; 1.44–8.46) participants exposed to whole-body vibration were at an increased risk of sciatica. Individuals of normal weight who were exposed to vibration, and overweight and obese individuals who were not exposed to vibration were not at an increased risk. The risk of hospitalization for sciatica seems to be highest among obese individuals exposed to whole-body vibration and among those lifting or carrying heavy objects.

## Introduction

Sciatica is a relatively common musculoskeletal disorder with a prevalence ranging from 2–5%, depending on the population^1–3^,. It is the cause of high health-related costs to society and a high disability burden to individuals suffering from sciatica^4,5^.

Earlier studies have found associations between occupational factors and herniated lumbar disc or sciatica. Knowledge regarding work-related factors and the risk of sciatica has not progressed much in recent decades. Many earlier studies have investigated work-related factors in a cross-sectional or case-control study design, which can cause recall bias in exposure to workload. In these study designs, participants with sciatica may have selected lighter work tasks and the ‘healthy worker effect’ may have influenced the results^6^.

Physical activity at work and occupational workload, such as lifting or carrying heavy objects, have been found to increase the risk of sciatica^1,7–12^, Non-occupational lifting, especially with straight knees and a bent back, has also been associated with an increased risk of herniated lumbar disc^13^, Work-related twisting of the trunk^14^, and occupational exposure to whole-body vibration (for example motor vehicle drivers, machine operators) have also been suggested to be risk factors for sciatica^8,15,16^, However, some prospective studies have disputed the role of physically heavy labour as a risk factor of sciatica^8,10,14,17^.

Obesity has previously been linked to sciatica, and a recent meta-analysis^18^, confirmed this. It is, however, unknown whether exposure to occupational workload factors increase the effect of excess body mass on sciatica.

In the present study, we assessed whether work-related factors predict hospitalization for sciatica in a longitudinal study design based on a national survey of Finnish adults. More specifically, we explored whether the physical strenuousness of an occupation, and specific strains – such as lifting and carrying heavy objects, awkward trunk postures, prolonged standing, prolonged sitting, whole-body vibration, constantly repeated series of movements and working speed determined by a machine (paced work) – were risk factors for hospitalization for sciatica.

## Materials and Methods

### Study population

The baseline data collection between 1978 and 1980 (the Mini-Finland Health Survey) is described in detail elsewhere (https://www.thl.fi/en/web/thlfi-en/research-and-expertwork/population-studies/finnish-mobile-clinic/mini-finland-health-survey). The original study population was a stratified, two-stage cluster sample representing Finnish adults aged 30 years or over. In the first stage, 40 representative areas were chosen. In the second stage, a systematic sample of residents was selected from each area. The original sample included 8000 participants (3637 men and 4363 women). A total of 7217 individuals (90%) participated in the screening phase of the study, which comprised questionnaires, interviews and laboratory tests. Those with history, symptoms or findings indicating a musculoskeletal disorder were invited to a clinical examination carried out by a specially trained physician.

Of the 5087 participants aged 30 to 59 years, 546 were no longer working, and 790 had a low back syndrome diagnosed by a physician during the clinical examination^19^, For 84 participants, their first hospitalization for sciatica preceded the baseline examination. A total of 1196 participants met one or more of the exclusion criteria. The cohort of the current study included 1900 men and 1991 women, who were followed up until December 31, 2011.

### Follow-up

The mortality and morbidity of the study population were followed up from the baseline examination by record linkage to nationwide registers. We obtained the data on hospitalizations for sciatica from the Care Register for Health Care, which covers all Finnish hospitals, public and private. The register is upheld by the National Institute for Health and Welfare, and diagnoses are based on the International Classification of Diseases (ICD). In the Eighth Revision, sciatica was defined by codes 353.99, 725.10 or 725.19; in the Ninth Revision by 7225A, 7227C or 7228C; and in the Tenth Revision by G55.1, M51.1, M51.2, M54.3 or M54.4.

### Work-related determinants

We used a baseline questionnaire to elicit information on present and previous occupations, and their overall physical strenuousness and specific exposures (https://www.thl.fi/documents/189940/3022770/MS011+Basic+questionnaire.PDF/f2e6deed-43f0-453f-a386-82824e3ad361). The questionnaire contained separate questions on the respondents’ longest-term work and their present work, but the minimum workload duration was one year. The following question determined the exposures: ‘Which of the following are (were) typical of your work: (a) lifting or carrying heavy objects, (b) stooped, twisted or otherwise awkward work postures, (c) continuous or almost continuous standing, (d) continuous or almost continuous sitting, (e) shaking of the whole body or use of vibrating equipment (for example working in a vibrating vehicle, operating a power saw), (f) a constantly repeated series of movements, (g) working speed determined by a machine?’ The response options were yes or no.

The participants classified the physical strenuousness of their work as (1) light sedentary work (work mainly consisting of sitting at a table, by a machine or controls etc. and involving only light manual work, for example intellectual work, study, sedentary office work, handling light objects), (2) other sedentary work (work that is mainly sedentary, but involves handling fairly heavy objects, for example, industrial work ‘at the conveyor belt’), (3) physically light standing work or light work involving movement (mostly standing work without cumbersome movements, or moving from one place to another without carrying heavy burdens, for example shop assistant work, crane operating, laboratory work, office work or teaching work that requires much moving about), (4) fairly light or medium-heavy work involving movement (work that largely involves moving about and a fair amount of stooping down and carrying, but not heavy burdens: this group also comprises work involving walking up and down stairs or fairly rapid motion for quite long distances, for example light industrial work, forest surveying, messenger’s work), (5) heavy manual work (either mostly standing work that involves much lifting of light objects or turning a crank etc., or lifting and carrying heavy objects, drilling, excavating, hammering etc., but with some sitting or standing, for example, work in the heavy engineering industry, construction work, using or assembling heavy tools, goods or parts, agricultural work using machines) and (6) very heavy manual work (work mostly consisting of continual or fairly continual heavy working movements, often with no interruption for long periods, for example carrying furniture, forest work, heavy non-mechanized agricultural work, fishing with heavy tackle, heavy construction work, excavation without machines). We also formed two dichotomous variables for the physical strenuousness of work: sedentary work involving handling fairly heavy objects, or physically light work (original Categories 2 and 3 versus all the other categories); and heavy or very heavy work (original Categories 5 and 6 versus all the other categories).

### Other determinants

We measured standing height and weight during the baseline examination, and calculated body mass index (BMI) as weight/height^2^ (kg/m^2^). For both genders, overweight was defined as a BMI of 25.0–29.9 kg/m^2^, and obesity as a BMI of ≥30 kg/m^2^. Body height was categorized into sex-specific tertiles: (1) 146–171 cm, (2) 172–177 cm and (3) 178–194 cm for men and (1) 117–159 cm, (2) 160–164 cm and (3) 165–180 cm for women. The participants were grouped into three classes according to their smoking habits: (1) never, (2) past or (3) current smokers. Leisure-time physical activity was elicited by the following question in the baseline questionnaire: ‘How much do you move about and how hard do you exert yourself physically in your leisure-time?’ and categorized as (1) little physical activity, (2) irregular leisure-time physical activity and (3) regular leisure-time physical activity. We classified self-rated general health according to a three-point scale: good, moderate and poor. We classified education level on the basis of the home interviews into three categories based on years of education: (1) <8 years, (2) 8–12 years and (3) >12 years.

### Statistical analysis

The follow-up period in the current study was from the baseline examination until the first hospitalization for sciatica; death; 65 years of age; or end of the follow-up period, December 31, 2011; whichever came first. We used the Cox proportional hazards regression model to analyse the associations between various risk factors and the incidence of sciatica leading to hospitalization. During the follow-up, the participants were censored after they died or turned 65. The confounding factors were chosen on the basis of the literature, and their inclusion was based on analyses in the current study population. In the analyses, we took into consideration age, sex, body height, body mass index (BMI), and smoking, which are considered risk factors for sciatica and could thus confound the associations.

We first performed analyses adjusted for age and sex. Second, we included the variables showing at least suggestive associations (P-value of < 0.5) with the risk of sciatica in the full model. Age was thus entered as a continuous variable, and the original six categories of the physical strenuousness of work were replaced by two dichotomous variables: sedentary work involving the handling of fairly heavy objects, or physically light work (original Categories 2 and 3 versus all the other categories); and heavy or very heavy work (original Categories 5 and 6 versus all the other categories). All the p-values were p-values for heterogeneity and based on likelihood ratio statistics (except the trend test for age in the multivariable analysis). We estimated hazard ratios (HRs) with 95% confidence intervals (CIs), and studied effect modification by entering all the multiplicative first-degree interaction terms of the potential risk determinants, one by one, into the full model. We used the likelihood ratio statistics to test the statistical significance of the associations and interactions. We used the SAS System for Windows, version 9.1 (SAS Institute, Inc., Cary, NC) in all the analyses.

### Ethical aspects

The Mini-Finland Health Survey was carried out prior to the current legislation on medical research on human subjects. The participants were fully informed of the use of the collected data for research purposes, and they participated on a voluntary basis, in compliance with the principles of the World Medical Association (WMA) Declaration of Helsinki. Agreeing to participate in the baseline health examination was taken to indicate informed consent.

## Results

For 75% of the 3891 cohort members, their present or last occupation was the longest in their work history, and the mean duration of that work was 17.0 (SD 9.5) years. For the rest, the mean duration of their longest occupation was 12.4 (SD 6.7) years, in their present or last occupation 4.6 (SD 3.3) years.

In total, 120 participants were hospitalized for sciatica during 111 416 person-years of follow-up. Until 1986 (Eighth ICD Revision), the most frequent ICD codes were 725.10 (28 cases, lumbar intervertebral disc displacement) and 353.99 (five cases, sciatica); between 1987 and 1995 (Ninth ICD Revision) they were codes 7227C (43 cases, intervertebral disc disease with myelopathy, Syndroma ischiadicum); and from 1996 onwards, codes M51.1 (24 cases, lumbar intervertebral disc disease with radiculopathy) and M54.3 (seven cases, sciatica).

### Age- and sex-adjusted analysis

Table 1 presents the distributions of baseline characteristics, and HRs with 95% CIs for hospitalization due to incident sciatica adjusted for age and sex. Current smoking was associated with an increased risk of sciatica. Compared with physically light sedentary occupations, other sedentary work involving the handling of fairly heavy objects increased the risk of hospitalization for sciatica (HR 2.72, 95% CI 1.36–5.44). Participants who rated their occupations as consisting of physically light moving/standing work were also at a higher risk of sciatica (HR 1.78, 95% CI 1.06–3.02). Physically heavier work seemed to protect against sciatica. From the reported specific exposures, either current occupation or that with the longest duration, comprising lifting or carrying heavy objects (HR 1.47, 95% CI 1.02–2.10), and exposure to vibration (HR 1.66, 95% CI 1.03–2.67) significantly predicted the risk of sciatica. Other exposures such as awkward trunk posture, prolonged standing or sitting, constantly repeated series of movements, or paced work showed no significant association with incident sciatica (Table 1).

**Table 1:** Baseline characteristics and their associations with incident sciatica. Hazard ratios (HRs) with 95% confidence intervals (CIs) adjusted for age and sex.

Characteristic	Sample	Event	HR, 95% CI	P value
**Age**				
30–39	1649	75	1.00	
40–49	1279	36	0.65 (0.43–0.96)	<0.001
50–59	963	9	0.23 (0.12–0.46)	
**Sex**				
Men	1900	65	1.00	0.13
Women	1991	55	0.78 (0.55–1.12)	
**Body height tertile**				
117–159 cm/146–171 cm	1312	31	1.00	
160–164 cm/172–177 cm	1327	45	1.21 (0.76–1.92)	0.71
165–−180cm/178–194 cm	1252	44	1.12 (0.70–1.79)	
**BMI** (**kg/m**^**2**^)				
<25	2034	63	1.00	
25–29.9	1400	42	1.13 (0.76–1.68)	0.43
≥30	457	15	1.47 (0.83–2.60)	
**Educational level**				
<8 years	2238	65	1.00	
8–12 years	1030	40	1.09 (0.73–1.63)	0.13
>12 years	623	15	0.62 (0.35–1.09)	
**Self-rated health**				
Good	2537	88	1.00	
Moderate	1162	27	0.89 (0.57–1.38)	0.78
Poor	191	5	1.25 (0.50–3.11)	
**Smoking**				
Never	2036	50	1.00	
Past	799	25	1.27 (0.77–2.08)	0.06
Current	1056	45	1.67 (1.09–2.55)	
**Leisure-time physical activity**				
Little	1117	37	1.00	
Irregular	2022	57	0.82 (0.54–1.23)	0.63
Regular	752	26	0.90 (0.54–1.48)	
**Physical strenuousness of work**				
Light sedentary	966	24	1.00	
Other sedentary	200	12	2.72 (1.36–5.44)	
Physically light	828	34	1.78 (1.06–3.02)	
Fairly light/medium-heavy	969	31	1.46 (0.86–2.50)	
Heavy	708	16	0.92 (0.49–1.75)	0.01
Very heavy	220	3	0.57 (0.17–1.90)	
**Lifting**				
No	2316	62	1.00	0.04
Yes	1575	58	1.47 (1.02–2.10)	
**Awkward trunk postures**				
No	2385	81	1.00	0.30
Yes	1506	39	0.82 (0.56–1.20)	
**Prolonged standing**				
No	2252	69	1.00	
Yes	1639	51	1.01 (0.70–1.45)	0.97
**Prolonged sitting**				
No	2597	73	1.00	
Yes	1294	47	1.17 (0.81–1.69)	0.40
**Vibration exposure**				
No	3296	93	1.00	0.04
Yes	595	27	1.66 (1.03–2.67)	
**Constant movements**				
No	3094	97	1.00	0.78
Yes	797	23	0.94 (0.59–1.48)	
**Paced work**				
No	3609	112	1.00	
Yes	282	8	0.89 (0.44–1.83)	0.75

Mini-Finland Health Survey 1978–1980. Follow-up via Care Register for Health Care until 2011.

### Multivariable analysis

Table 2 shows the full multivariate model with HRs and their 95% CIs for risk of hospitalization due to sciatica. Current smoking (HR 1.49, 95% CI 0.97–2.28) and frequent lifting (HR 2.10, 95% CI 1.35–3.26) were associated with an increased risk of hospitalization for sciatica. Sedentary work involving the handling of fairly heavy objects/physically light work showed an increased risk of hospitalization for sciatica (HR 1.57, 95% CI 1.05–2.34), whereas heavy/very heavy work predicted a reduced risk (HR 0.48, 95% CI 0.26–0.89).

**Table 2:** Full model hazard ratios (HRs) and their 95% confidence intervals (CIs) of incident sciatica.

Characteristic	HR	95% CI	P value
Age	0.59	0.47–0.73	<0.001
Sex, women vs. men	0.87	0.57–1.35	0.54
**BMI** (**Ref**. **group: > 25 kg/m**^**2**^)			
25–29.9	1.13	0.75–1.70	
≥30	1.39	0.78–2.48	0.53
**Smoking** (**Ref**. **group: never**)			
Past	1.20	0.73–1.98	
Current	1.49	0.97–2.28	0.19
**Educational level** (**Ref**. **group: < 8 years**)			
8–12 years	1.16	0.76–1.75	0.30
>12 years	0.72	0.39–1.34	
Sedentary work involving handling fairly heavy objects/physically light work	1.57	1.05–2.34	0.03
Heavy/very heavy work	0.48	0.26–0.89	0.02
Lifting	2.10	1.35–3.26	0.001
Awkward trunk postures	0.68	0.43–1.03	0.06
Prolonged sitting	1.14	0.76–1.71	0.54
Exposure to whole-body vibration	1.61	0.95–2.72	0.08

In the model, age is continuous (HR per one SD, 8.4 years of age). All risk factors are dichotomized except for Body Mass Index (BMI) and smoking. Mini-Finland Health Survey 1978–80. Follow-up via Care Register for Health Care until 2011.

### Effect modification

An interaction between excess body mass and exposure to whole-body vibration occurred, which affected the risk of hospitalization for sciatica (Table 3). Overweight and obese participants exposed to whole-body vibration at work were at a significantly increased risk of hospitalization for sciatica, whereas overweight or obese participants not exposed to vibration, and normal weight participants exposed to vibration were not at an increased risk.

**Table 3:** Joint effect of body mass index and exposure to whole-body vibration at work on risk of hospitalization for sciatica.

Exposure to vibration	Body Mass Index (kg/m^2^)	Sample	Event	HR	95% CI
No	<25	1779	56	1	
Yes	<25	255	7	0.85	0.36–1.99
No	25–29.9	1146	29	0.96	0.61–1.53
Yes	25–29.9	254	13	1.94	0.96–3.93
No	≥30	371	8	0.93	0.44–1.97
Yes	≥30	86	7	3.50	1.44–8.46

*P-value for the interaction term of body mass index and exposure to vibration was 0.05.

Hazard ratios (HRs) with 95% confidence intervals (CIs) are adjusted for age, sex, height, smoking, leisure-time physical activity, physical strenuousness of work, lifting, awkward trunk postures, prolonged standing, prolonged sitting, constantly repeated series of movements, and paced work. P-value 0.05.

## Discussion

The prevalence^3^, and determinants^19^, of sciatica and other low back syndromes in the Mini-Finland Health Survey were reported a generation ago in a cross-sectional design. Our current cohort study was based on this survey. The present study indicates that lifting or carrying heavy objects at work, sedentary work involving the handling of fairly heavy objects/physically light standing or moving work, and exposure to whole-body vibration predicted incident sciatica leading to hospitalization during the 30-year follow-up. Heavy/very heavy work predicted a reduced risk of hospitalization for sciatica. Of the more defined exposures, a history of lifting or carrying heavy objects at work predicted an increased risk of hospitalization for sciatica. Some factors significantly modified each other’s effects: obese participants exposed to vibration at work were at a significantly higher risk of being hospitalized for sciatica, whereas BMI or vibration alone showed no prediction.

Earlier studies have mainly observed that physically heavy work increases the risk of sciatica^1,8,10,17^, However, the same has not been found among Finnish middle-aged employees^20^, In the current study, sedentary work involving handling fairly heavy objects, or physically light work involving standing or moving predicted incident sciatica, whereas heavy/very heavy physical work even protected against hospitalization for sciatica. Individuals in physically poorer condition may seek lighter work tasks, which would explain the increased risk in these work tasks. On the other hand, participants who can endure physically heavy occupations probably have better physical and muscle condition, which would enable better neuromuscular control of the spine and trunk. One possible explanation for the reduced risk of hospitalization for sciatica may be the better tolerance of the tissues among subjects who remain in physically heavy work tasks. In many occupational studies, the ‘healthy worker effect’ may also influence results, since unhealthy individuals may be excluded from the workforce^6^.

We found no association between obesity and hospitalization for sciatica, whereas obesity and exposure to vibration at work together increased the risk of sciatica. Earlier studies have suggested that overweight and obesity are associated with sciatica^18^, One reason for not finding such an association may be that we excluded the non-working population from the analysis. Unemployed people are more likely to be overweight/obese than the working population.

Earlier studies have found sitting to be associated with the emergence of low back disorders^15,21^, However, only a minority of such studies have focused on sciatica, and many have analysed the prevalence of sciatic pain in occupations in which sitting is a major requirement but in which workers are also co-exposed to awkward postures and vibration^22–25^, Intradiscal pressure seems to be higher while sitting and this may be one of the underlying mechanisms contributing to sciatica in sedentary occupations^26^, In the current study we found that sedentary work which also involves handling fairly heavy objects predicted the risk of hospitalization for sciatica, whereas sitting exposure *per se* did not.

In line with previous studies^8,13,27^, we found that lifting and carrying heavy objects predicted a risk of sciatica leading to hospitalization. Lifting and carrying have been found to increase intradiscal pressure^28,29^, and to predispose discs to segmental structural failure. In our study questionnaire, one category of the physical strenuousness of the work included lifting and carrying heavy objects, while it was simultaneously analysed as a specific exposure. This may have caused overlapping and affected our results, although independent parts of our questionnaire obtained information on general strenuousness and specific exposures at work.

Earlier studies have found associations between vibration exposure and back pain^22–25^, However, most of these studies have focused on low back pain; only a few have focused on sciatica. Nevertheless, there is clear evidence of an increased risk of sciatic pain with exposure to vibration^7,16,22,25^, Occupational exposure to vibration seems to be particularly deleterious. Burström *et al*. found that employees exposed to vibration were at approximately a two-fold risk of sciatica or low back pain compared to non-exposed workers^30^, It has been suggested that vibration is a significant co-exposure, and workers who are exposed to vibration while sitting during their work tasks are at a higher risk of sciatica^31^, In our study, vibration exposure did not statistically significantly predict hospitalization for sciatica in the full multivariate model. Only overweight or obese individuals exposed to vibration at work were at a higher risk of sciatica leading to hospitalization. The harmful effect of vibration exposure on intervertebral discs may be augmented among obese participants. This could offer an interesting preventive aspect in occupational health care, if replicated.

In line with previous studies^31–33^ current smoking at baseline predicted incident sciatica. It has been suggested that smoking can cause sciatica by interfering with the nutrition of the intervertebral discs^33,34^ and/or increasing the production of pro-inflammatory cytokines in the intervertebral discs^35^.

Our study was based on a nationally representative sample and had a very high participation rate. The outcome was based on international ICD codes, and we obtained the data on hospitalizations for sciatica from the Care Register for Health Care, which is known to be a reliable and accurate source^36^. This register also includes diagnoses from day surgery treatments. The follow-up used national registers with excellent coverage, which implies that our results have no significant bias. Thus, our findings can be generalized to the overall population. This is an obvious strength of the study, due to both the representativeness of the base follow-up population and the validity of the associations. Another strength is that we were able to exclude participants with a history of back pain at baseline.

This study also has limitations. Not every patient with sciatica is hospitalized, but in our study the risk factor may have predicted incident sciatica, likelihood of hospitalization once sciatica occurred, or both. During the follow-up period of our study, not every patient suffering from sciatica was hospitalized, due to natural causes of sciatica and changes in the treatment methods over the decades. Therefore, our study partly investigated the reasons why people develop sciatica. The coverage of the Care Register for Health Care has improved in recent years to more than 95%^36^, Due to long follow-up times, admission and referral to hospital may have varied over the decades and the content of the hospital treatment for sciatica has also changed. For example, earlier, in-patient bed rest was recommended, but today, staying active and out-patient disc surgery are preferred.

The information on working conditions and exposures was based on questionnaire data and no objective measurements were taken, which may be an obvious limitation of our study. Objective measurement would have been the preferred method, but this was not feasible in such a large study. The questionnaire did not elicit income level, so we were not able to take into account the effect of income level on the risk of hospitalization for sciatica in this study, which is also a limitation. As the follow-up period was so long, it is possible that the work tasks and occupations of the respondents changed. This may also have affected our results.

## Conclusions

The risk of hospitalization for sciatica seems highest among overweight or obese individuals exposed to whole-body vibration, among those lifting or carrying heavy objects, or among those in sedentary occupations involving the handling of fairly heavy objects. In contrast, we found heavy/very heavy work to be a protective factor. The effect-modification of obesity on vibration exposure which we found in the current study must be replicated before any causal inference or preventive application is justified.
